# Overcoming Barriers: Adolescents’ Experiences Using a Mobile Phone Dietary Assessment App

**DOI:** 10.2196/mhealth.5700

**Published:** 2016-07-29

**Authors:** Åsa Svensson, Maria Magnusson, Christel Larsson

**Affiliations:** ^1^ Department of Food and Nutrition Umeå University Umeå Sweden; ^2^ Angered Hospital Västra Götaland Region Sweden; ^3^ Section for Epidemiology and Social Medicine Sahlgrenska Academy University of Gothenburg Gothenburg Sweden; ^4^ Department of Food and Nutrition, and Sport Science University of Gothenburg Gothenburg Sweden

**Keywords:** adolescents, content analysis, dietary assessment, Self Determination Theory, mobile phone app

## Abstract

**Background:**

The use of new technology has the potential to increase participation rates in dietary studies and improve the validity of collected dietary data. However, to evaluate the usability of developed dietary methods, qualitative studies of participants’ experiences and perceptions are needed.

**Objective:**

To explore adolescents’ experiences using a newly developed mobile phone dietary assessment app, with a focus on factors that could affect their recording of dietary intake.

**Methods:**

Focus group interviews were conducted with 75 participants who had used a newly developed mobile phone dietary assessment app in a quantitative evaluation study. The interviews were analyzed using qualitative content analysis and the theoretical framework of Self Determination Theory was applied.

**Results:**

The adolescents’ use of the mobile phone dietary assessment app was characterized by their struggle to overcome several perceived barriers. Facilitators that helped adolescents complete the method were also identified. Motivation was found to be an important facilitator, and intrinsically motivated participants completed the method because they found it fun to use. The autonomous extrinsically motivated participants completed the method for the greater good, in order to contribute to the study. The controlled extrinsically motivated participants completed the method to get a reward or avoid punishment. Amotivated participants did not complete the method. More motivated participants were assumed to be more able to overcome barriers and needed less facilitators.

**Conclusions:**

Future studies that examine the recording of food intake should include systematic efforts that aim to minimize identified barriers and promote identified facilitators. Further research should specifically aim at studying methods for (and effects of) increasing intrinsic motivation by supporting autonomy, competence, and relatedness among adolescents asked to participate in dietary studies.

## Introduction

Studies that examine the associations between diet and health outcomes require methods of dietary assessment that correctly assess food intake during the time period of interest [1]. Furthermore, there is a need for improved dietary assessment methods that are accepted by study participants. The food record (FR) is a commonly used method to assess individual dietary intake for one or several days, which has the advantage of not being dependent on the respondent’s memory [2]. However, the burden of recording all consumed foods can lead to altered dietary intake, and some foods may be omitted from the FR. Consequently, the assessed dietary intake will be either unrepresentative or underestimated, representing a widespread problem when using FR and other dietary assessment methods [3].

The assessment of dietary intake is challenging in all age groups and especially among adolescents [4]. Studies have suggested that adolescents are less accurate reporters of their dietary intake compared to young children and adults due to less structured eating habits, a relatively large proportion of meals consumed outside of the home, or lack of motivation to participate in dietary studies [4,5]. It is therefore important to improve dietary assessment methods in this age group.

Children’s and adolescents’ views of keeping an FR have been investigated using qualitative methodology, and it was found that those aged 12 years and older were reluctant to keep FRs [6]. One reason for this was that adolescents did not want to carry a paper FR and portion size booklet while they were with their peers. Respondents also expressed that they would consider changing their dietary intake to avoid recording, suggesting a need to make the process of keeping an FR less burdensome. One study found that adolescents preferred using technology-based FRs (ie, camera or personal digital assistant) versus a traditional pencil and paper FR [5]. The widespread use of smartphone technology has led to new possibilities in dietary assessment.

In Sweden, 89% of adolescents aged 13-16 years own an advanced-feature mobile phone [7]. Using a mobile phone to keep an FR is one way to adapt the dietary assessment method to adolescents’ lifestyles, and to circumvent the need to bring a traditional pencil and paper FR to school and friends’ homes. No evaluated mobile phone FR method was available in Sweden to make it more convenient for adolescents to record dietary intake, so we developed a mobile app in a previous study [8]. To evaluate the feasibility of the newly developed method, there was interest in exploring not only quantitative parameters indicating its validity, but also the users’ experiences and views of using the Swedish mobile phone dietary assessment app.

The aim of the present study was to explore adolescents’ experiences using a newly developed mobile phone dietary assessment app, with a focus on factors that could affect their recording of dietary intake.

## Methods

### Participants and Setting

This study includes adolescents who participated in an evaluation study of a newly developed mobile phone dietary assessment app during 2013. Participants were recruited by visits to schools in the city of Göteborg and neighboring municipalities in Västra Götaland, Sweden. The evaluation study has been described in a previous paper [8]. A total of 389 adolescents in 28 school-classes were given information about the study during a first visit in class; 148 of whom chose to participate in the quantitative part of the evaluation study (47 during spring term and 101 during autumn term). In the qualitative part of the study, twelve group interviews (three during the spring term and nine during the autumn term) were performed. Teachers made it possible to assign class time for interviewing 92 of the 148 adolescents, and at the time of the interview 17 students were not in school. Thus, group interviews were conducted with a total of 75 participants.

### Ethical Considerations

This study was approved by the Regional Ethical Review Board in Umeå, Sweden. The adolescents were informed about the aim of the study, and were told that participation was voluntary and all collected data would be treated with confidentiality. All participants gave written informed consent, and for adolescents younger than 15 years of age, a parent additionally gave written informed consent.

### Pilot Test of Procedures and Methods

A pilot test was conducted in November 2012 to test all methods included in the evaluation study, and to practice the study procedures. The pilot test took place in a school on Orust, an island located one hour from Göteborg, with five girls and one boy from one school-class. A group interview was conducted to explore possible improvements that could be made in the procedure of data collection and the mobile phone dietary assessment app itself, as well as to practice the interview procedure. The pilot test did not lead to any fundamental changes to the app, but revealed some technical problems that needed to be solved.

### Procedures and Methods in the Quantitative Part of the Evaluation Study

**Figure 1:**
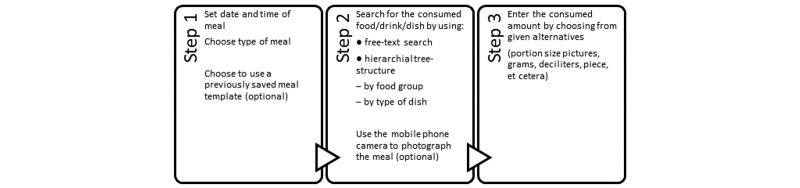
The steps involved in registering dietary intake in a newly developed Swedish mobile phone app. The three steps are repeated until all foods and drinks in a meal have been entered, and the meal is thereafter saved and automatically sent to a server for storage and calculation of energy and nutrient contents.

The mobile phone dietary assessment app has been described in detail [8], and the procedure of recording dietary intake in the app is illustrated in Figure 1. In summary, the first step was to enter the date and time of the meal (with the current date and time as default) and type of meal (breakfast, lunch, dinner, or snack). Thereafter, the user searched for the consumed food/drink/dish in a food database by using free text search, and choosing from a food group category or type of dish. The app uses the Swedish national food database, which includes over 1900 foods, drinks, and dishes. The amount consumed was thereafter entered by choosing from portion sizes (eg, in gram, deciliter, table spoon, tea spoon, or piece) that were given as alternatives for each food/drink/dish. For several items there were also pictures of foods of known weight and increasing portion size to aid the estimation of consumed amounts. After all foods in a meal had been entered, the meal was saved and automatically sent to a central server for storage and calculation of energy and nutrient contents. The saved meals could be accessed in the app through an archive of registered days, in which entered foods and amounts could be changed if necessary.

In addition to recording dietary intake, the user was asked to answer eight questions in the app every evening. The questions pertained to the use of dietary supplements, the approximate percentage of the dietary intake that was recorded, the physical activity level during the day (out of five predefined levels), the level of dietary intake and physical activity (higher or lower than normal), whether the user had tried to gain or lose weight during the day, and if the user had felt stressed or anxious. Users had access to feedback about the dietary intake (energy, fruits and vegetables, macronutrients, and five micronutrients) in relation to recommended daily intakes [9], as well as total energy expenditure calculated from the reported daily activity level, basal metabolic rate [10], and body mass index. The study participants were encouraged to eat as usual and not change their intakes based on the feedback. Additional functions in the mobile phone dietary assessment app were to receive reminders (status bar notifications) to register with a chosen time interval, and to save a meal as a template to be loaded the next time the same meal was consumed. The app was connected to the mobile phone camera and the user could take a picture of their meal as a memory aid if the consumed foods could not be entered until later.

Participants in the quantitative part of the study were asked to record all foods and drinks consumed for three consecutive days using the mobile phone dietary assessment app. Respondents were also asked to answer the in-app questions in the evenings, during the same days as recording their dietary intake. Those who did not have an Android mobile phone (72/81; 89% of the participants who completed the quantitative part of the study) borrowed a phone with data traffic subscription and a charger, and were given an instruction manual on how to use the mobile phone. Those who had their own Android mobile phone were given instructions, and help to download and install the app on their own phones.

Additional measurements in the evaluation study included the SenseWear Armband (BodyMedia, Inc.; Pittsburgh, PA, USA) for registration of total energy expenditure during the same days as recording dietary intake, anthropometric measurement of weight and height, and a questionnaire aiming to measure factors that could possibly influence the accuracy of reported dietary intake. Participants in the spring term also recorded dietary intake using a web-based FR. However, due to high perceived participant burden, participants in the autumn term were not asked to complete the additional three days of dietary recording using the web-based FR. During the first part of the autumn term, participation was still low and it was decided that a cinema ticket would be used as an incentive that was given to the participants who completed all methods.

### Focus Group Interviews and Data Collection

A second in-class visit was made after one to two weeks, when the adolescents had finished the quantitative part of the study. The adolescents were then asked to participate in a focus group interview that examined how they perceived the use of the mobile phone dietary assessment app. Semistructured interviews were performed with groups of 4-9 participants. Teachers provided rooms in which the interviews could take place undisturbed. The interviews were audio recorded after consent from the participants, who were ensured that the material would be treated with confidentiality and that no one except the researchers involved in the study would have access to the recordings and transcripts. The first author conducted the interviews using an interview guide that included questions related to the study aim (see Multimedia Appendix 1). The interview guide consisted of 20 questions that were grouped into introductory questions, key questions, and concluding questions. The guide was used as an aid to ensure that the interviews focused on relevant topics, and was not followed to the letter. The questions were used to initiate a discussion, and the participants were free to elaborate on the topics and introduce topics of their own interest, in relation to the study. In seven of the twelve interviews, an assistant was present to take notes and occasionally ask questions. Participants in three of the interview groups had used the web-based FR, and participants in seven of the groups had been offered cinema tickets if they completed all methods in the quantitative part of the study.

### Data Analyses

The interviews were transcribed verbatim and data were analyzed inductively using the principles for qualitative content analysis according to Graneheim and Lundman [11]. The first and second author performed the analysis separately, and thereafter discussed and agreed on the findings. Each transcribed interview was treated as a unit of analysis. The transcripts were read several times and meaning units, with content related to the research questions, were identified and condensed. Thereafter, codes were applied to the condensed meaning units. After the spring term, the first three interviews were coded, and after the autumn term the remaining nine interviews were coded. Codes were then compared and changed if necessary, so that one set of codes fit the entire collection of materials. Thereafter, the codes were sorted into categories that were exhaustive and mutually exclusive [11]. Examples of condensed meaning units, codes, and categories can be found in Table 1. The original spoken language in the interviews was Swedish. After the analysis had been completed, the results were translated into English.

**Table 1:** Examples of meaning units, condensed meaning units, codes, and categories.

Meaning unit	Condensed meaning unit	Code	Category
You always have the phone with you anyway, so it is only to record directly	You have the phone with you all the time and can record directly	Advantage with mobile phone	Benefits of using the mobile phone app
Sometimes I felt that there were too many differences; if you searched for butter and milk there were different types so I just wrote milk. You have no idea which one to choose	Sometimes there were to many different types of butter and milk; you have no idea which one to choose	Hard/difficult to choose between many alternatives	Recording correctly
You thought more about it; now I will eat a snack because now I will be the good type	You thought of what you ate, eating a snack to be good	Improve dietary intake	Dietary intake was affected

### Theoretical Framework

Many motivational theories exist, and according to Self Determination Theory (SDT) there are three basic psychological needs for an individual to function well: autonomy (a sense of choice and freedom from external pressure), competence (the ability to master a task and understand the rationale behind it), and relatedness (the need to belong and feel connected to others) [12]. Social contexts may support these three needs and thus result in better persistence and performance on activities. Consequently, when these needs are satisfied, motivation is enhanced.

For the present study, the motivational types of SDT described by Wenemark [13] were applied. Rather than categorizing the participants according to the motivational types on an individual level, we tried to identify the different motivational types among the participants to see if examples of all types could be found. SDT categorizes motivation into six groups: intrinsic motivation, four types of extrinsic motivation, and amotivation. Intrinsic motivation is a natural inclination to explore and learn, and is characterized by spontaneous interest and enjoyment in an activity [12]. Extrinsic motivation can be integrated to different degrees, or can be self-determined, and locus of causality can be perceived as external or internal. In the two types of extrinsic motivation characterized by external locus of control, actions are controlled by others or by the self through rewards or punishments [12]. Wenemark [13] named these motivational types *controlled extrinsic motivation*. In the two categories of extrinsic motivation characterized by internal locus of control, actions are of importance for the self and possibly internalized. These motivational types were named *autonomous extrinsic motivation* by Wenemark [13]. Amotivation is a complete lack of intention to act [12].

## Results

### Characteristics of the Participants

The 92 participants in the school-classes in which group interviews were conducted were 14-16 years of age and spoke fluent Swedish. Most participants (71/92, 77%) were normal weight, 59% (54/92) were girls, and 49% (45/92) had at least one parent with a university/college education. This finding was comparable to the 148 adolescents who participated in the quantitative part of the evaluation study. The schools and municipalities in which recruitment took place were comparable regarding the proportion of adolescents with a foreign background (adolescents and/or both parents born outside of Sweden). Of the 92 adolescents, the proportion with at least one parent with a university/college education was smaller (45/92, 49%) than the proportion of adolescents with at least one parent with higher education in the municipalities (63%). The participants were not identified at the time of the group interviews, so characteristics of the 75 who participated are not available separately from the 92 adolescents in the school-classes in which the interviews were conducted.

The interviews lasted between 12 and 29 minutes (average time 19 minutes). More time had been earmarked for the interviews; however, the participants had said what they wanted to leave before the time had run out.

### Categories

**Figure 2:**
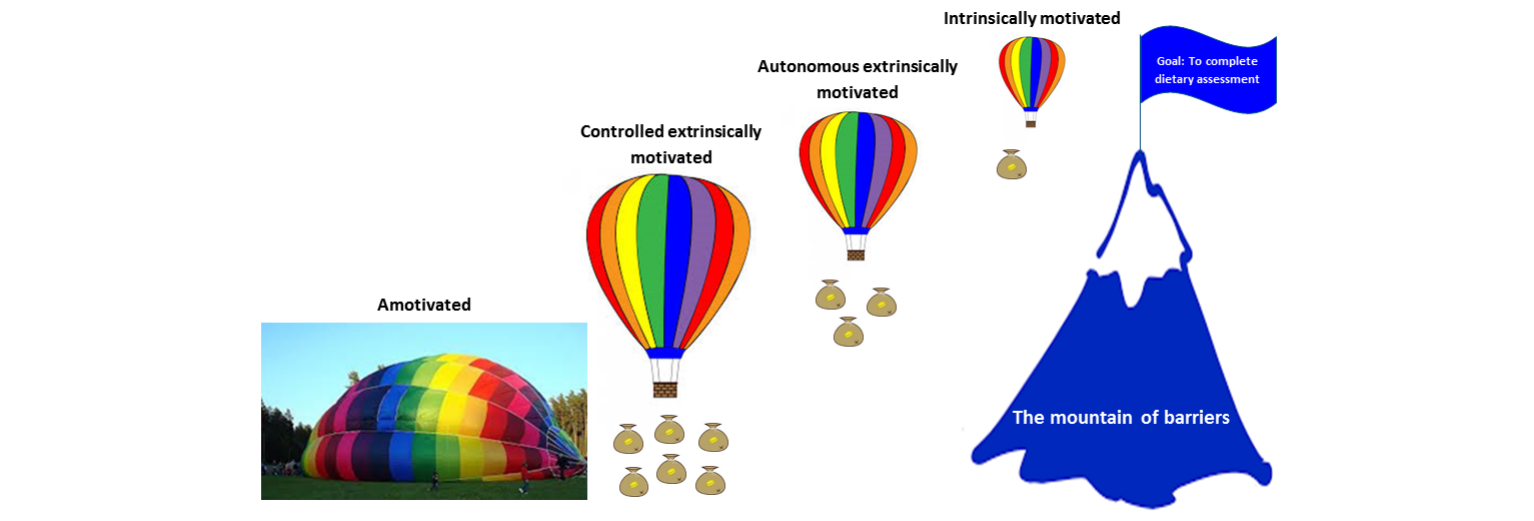
Illustration of adolescents' experiences of using a mobile phone dietary assessment app. To reach the goal of a complete dietary registration at the top of the mountain, participants must overcome several perceived barriers. Depending on their motivational type, subjects are in a smaller or larger balloon, and therefore need less or more facilitators (sandbags to let go of) to make it rise.

Categories illustrating participants’ views of recording dietary intake with the mobile phone dietary assessment app were grouped into seven categories, consisting of 43 codes (Table 2). An underlying theme in the material was identified as *To overcome the barriers* (Table 2, Figure 2). The theme should be interpreted as a thread of meaning that runs through the entire material [11]. In the following text, the manifest content is presented for the seven categories, followed by the theme.

**Table 2:** Codes, categories, and theme from qualitative content analysis of 12 group interviews.

Code	Category	Theme
*The design of the app matters* *The app was difficult* *There were technical problems with the app/mobile phone* *Unclear with feedback* *Unclear/frustrating with reminders* *Unnecessary functions* *Is the feedback correct?* *Difficult to find the feedback* *Difficulty with the questions in the evening* *Type of mobile phone matters* *Wants to record exercise*	Difficulties of using the mobile phone app	To overcome the barriers
*App is simple/good/fun* *Good and interesting with the results* *Good with the questions in the evening* *Advantage with mobile phone*	Benefits of using the Mobile phone app	
*It is easy to forget* *Easier to record in the evening* *Difficult to record (in general)* *Combines techniques to remember* *You do not give up* *You have to think about when you eat and when to record* *You get tired* *Impractical sometimes* *The recording was fun/OK*	The process of recording	
*Good with pictures* *Good selection of foods* *There is a need for more composite dishes* *Had to take something similar* *Difficult to record food with lots of ingredients* *Difficult to record sandwiches* *Difficult and unnecessary to record small meals* *Difficult/hard to estimate amounts* *Unclear what should be recorded* *Foods/dishes were missing* *Hard/difficult to record if you do not know content/type* *Hard/difficult to choose between many alternatives*	Recording correctly	
*Improve dietary intake* *Avoided eating so you do not have to record*	Dietary intake was affected	
*Reflects over the diet* *The days of recording were not representative*	Awareness of diet and physical activity habits	
*Lack of interest* *Unclear with the study/methods* *Worry about focus on weight and dietary habits*	The study is not for me	

#### Difficulties of Using the Mobile Phone App

*Difficulties of using the mobile phone app* were, according to participants, mainly technical and related to the app or type of mobile phone used. Many of the participants had to borrow an Android mobile phone for the study. Those who were not familiar with Android phones sometimes had difficulties using the phone, and participants stated that it would have been easier for them if the app had been available for iPhone.

Other difficulties that were mentioned concerned the design of the app, unnecessary functions, unclear reminders to record, the questions in the evening, and the feedback function. Some participants also said that they would have preferred to register exercise instead of answering a question about the physical activity level during the day.

#### Benefits of Using the Mobile Phone App

In contrast to the above, some participants thought it was beneficial to answer the questions in the evening since it gave them a good summary of the day, and they appreciated getting feedback about their dietary intake in the mobile phone dietary assessment app.

Other *benefits of using the mobile phone app* were that it was fun and easy to use. Participants liked the idea of using the phone, which they claimed to have with them all the time.

many usually take photos of their food and things, and put these out on the Internet; so it’s just to have an app and record what you eat.

#### The Process of Recording

Participants found the study interesting and fun, but there were also views that the recording of dietary intake was burdensome and that one could get tired of it after a while. Some participants expressed that even though the study could be experienced as burdensome, it only lasted for three days, which was not difficult to endure, and they did not want to give up. Some participants found it easier to record in the evening (eg, when they used a borrowed mobile phone), thus avoiding carrying two phones during the day. Some participants combined techniques (ie, took photographs or notes about the consumed foods using their own mobile phone and entered it in the app in the evening). Other important factors related to *the process of recording* include that it was easy to forget to record dietary intake, that one had to be constantly aware of what was consumed, and that it was sometimes impractical to record the foods consumed.

#### Recording Correctly

As presented in Figure 1, the process of *recording correctly* in the mobile phone dietary assessment app consisted of several steps, from knowing what type of food was consumed to making sure that the correct amount was entered in the app. For these steps, the design and user-friendliness of the mobile phone dietary assessment app (as well as other factors) were important. Participants sometimes found it difficult to know the type of food consumed or the content of a composite dish (eg, lunch in the school canteen when someone else had prepared the food). Another problem was how to correctly record a dish that consisted of several ingredients.

Tacos were very difficult to record because there was ground meat, spices and then all the other ingredients.

It was suggested that the mobile phone dietary assessment app needed more composite dishes. Some foods or dishes were not found when searched for, and participants had to choose something similar, although they were not always happy with the substitute and pointed out that it did not have the same nutritional content as the foods they had consumed. Despite this, some participants were surprised by the number of foods and dishes in the database, and that they also could find *foreign foods*. Interestingly, the opposite could also be perceived as a problem: there were too many alternatives for some foods (eg, cheese, bread, butter, and milk).

Sometimes I felt that there was too much difference; if you searched for butter and milk there were different types so I just wrote milk. You really have no idea which one to choose.

Participants found it tiresome and unnecessary to record snacks and small meals. Some stated that they did not record every snack. Sandwiches, which are a common meal in Sweden, were problematic in this study, since participants had to know and search for the correct type of bread, butter, cheese, or other spread. Defining the consumed amount was also pointed out as difficult. However, the portion size pictures were found to be helpful. Finally, there was some uncertainty regarding what foods needed to be recorded (eg, whether snacks and drinking water should be entered).

#### Dietary Intake was Affected

Some participants stated that they ate as usual and recorded everything they ate. However, in spite of the fact that the participants were told to eat as usual during the study, *dietary intake was affected* emerged as a category. The major influence was on those participants abstaining from their usual snacking to avoid having to enter the foods, and participants who stated that they consciously or unconsciously *improved* their dietary intake.

I sometimes did not eat things because then I would have to record them.

#### Awareness of Diet and Physical Activity Habits

Participants showed *awareness of their diet and physical activity habits* by questioning whether the days of recording were representative, as they considered that they had not exercised or eaten as usual during the study. This issue was especially true for those who participated during the weekend. After being in the study, some participants expressed that they had gained new insights about their dietary intake, and some were surprised by the results (eg, that the diet during the weekend was so *bad*, or that their energy intake was not higher).

#### The Study Is Not for Me

One participant asked if the purpose was to test the app or to assess their diet, even though it had been stressed that the aim was to evaluate the mobile phone dietary assessment app, and that no focus would be on individual diets when recruiting the participants. The purpose of the study was not clear to everyone, and some participants found that *the study was not for them*. Within this category, worries about body weight were a potential barrier to participating in the study. In one interview, participants said that the adolescents who decided not to participate did so because they were afraid that there would be a focus on their diet, physical activity, and body weight.

There were many who were afraid to become aware: “how bad I am, and I’m so bad for eating all this” or like… “I really should lose some weight”

Some participants stated that they joined the study in order to avoid other tasks assigned by the school teacher, thereby showing a lack of interest in the study even if agreeing to participate.

### Category Interpretation

Several barriers to keep a correct FR can be discerned in the categories described above. In Figure 2, barriers are illustrated as a mountain, which the participant must ascend to complete the FR. The barriers include perceived difficulties with handling the mobile phone and app, and the app not working as it should. These issues were obviously problematic for the completion of the study. The problems with the mobile phone dietary assessment app not working properly arose later during the study, and the source of these technical problems was not detected. Other barriers were related to recording the correct food and amount in the app, and were often related to the food database. Barriers also included the effort needed to keep an FR, such as keeping the app in mind when participants were unmotivated to do so, or were busy with other things. Furthermore, uncertainties about what to record (and why) made it more difficult for some participants. Barriers for an accurate reporting of the diet arose when participants changed their intakes because of the study.

In contrast to these barriers, there were also facilitators for using the mobile phone dietary assessment app and completing the FR. In Figure 2, facilitators are illustrated with sand bags that the participants are able to release in order to let the air balloon rise. Facilitators included notions that the app was easy to use and that it was fun and interesting to record dietary intake, use the methods, and see the results. Participants thought it was a good idea to use a mobile phone for the task, and if they did not succeed in using it during the day, they found their own solutions by combining techniques (eg, by using the camera function on their own mobile phones) and recording in the evening. Furthermore, participants thought that the food database had enough foods to correspond to their diets, and if they did not find an exact match they could choose something similar. Entering the correct amounts was facilitated by the portion size pictures. Finally, the participants’ determination to carry through facilitated their completion of the study.

### Theme

When considering the participants’ experiences and perceptions of using the mobile phone dietary assessment app, the theme of *overcoming barriers* became apparent (Table 2, Figure 2). It was burdensome to keep an FR and wear the SenseWear Armband, but even so, many participants were able to complete the task. Some participants admitted to getting tired of using the methods, but they still did their best to finish the study.

Motivation was an important facilitator in this study, and the motives to participate differed among participants. When applying the different types of motivation in SDT to the results of the present study, amotivated participants would be the only subgroup in the study that lacked motivation to use the mobile phone dietary assessment app, and fail to complete the FR. This subgroup is illustrated in Figure 2, with the participants lacking the necessary means (a balloon in flight-worthy condition) to overcome the barriers and reach the goal of a complete and correct FR on the top of the mountain. Controlled extrinsically motivated individuals would include participants who were facilitated by the motivation to get a reward or avoid punishment. In Figure 2, the controlled extrinsically motivated participants are in possession of a large balloon and need several facilitating factors to make it rise. Some participants stated that they were not interested in the study, but decided to participate in order to get a cinema ticket. Other respondents said that they participated so they would not have to go for a walk, which one teacher suggested as an alternative activity for students that were not recruited to the study. During the interviews conducted in spring and beginning of autumn, participants said that they would have liked some sort of reward for being in the study, as they lacked other motives to participate. In addition to the question of being rewarded or not, participants found the design of the app important, and some said that a more stylish app would motivate them more to use it.

Using the terminology of SDT, the autonomous extrinsically motivated participant completed the FR for *the greater good* to contribute to the study, and the intrinsically motivated participant enjoyed using the mobile phone dietary assessment app and completed the FR because it was fun. The second smallest and the smallest balloons in Figure 2 belong to the autonomous extrinsically motivated and the intrinsically motivated participants, respectively. The smaller the balloon, the less facilitators are needed to reach the top of the mountain (Figure 2). In the present study, some respondents said that they wanted to participate irrespective of rewards, as they found the procedures and results of the study fun and interesting (ie, they were more autonomous and perhaps even intrinsically motivated). One adolescent claimed that she chose to participate since not many of her classmates participated, and she felt sorry for the researchers, which could be considered autonomous extrinsic motivation. Furthermore, the participants differed in their interests in diet and health, which could explain differences among participants with autonomous extrinsic and intrinsic motivation, and their ability to overcome the barriers.

## Discussion

The goal of an FR is to obtain a correct record of all consumed foods and drinks, as well as the correct amounts during the day(s) of recording [2]. Furthermore, the dietary intake should not be changed as a result of keeping an FR. Using technology such as mobile phones in dietary assessment could facilitate the collection of valid dietary data. The present study aimed to explore adolescents’ experiences using a mobile phone dietary assessment app, with a focus on factors that could affect their recording of dietary intake. The results generated the theme *To overcome the barriers*. Even though the mobile phone dietary assessment app had the potential of being fun and easy to use, there were difficulties with the method, and the end result depended on whether or not the participants were able to overcome the barriers with the help of facilitating factors. The adolescents’ motivation to continue recording their dietary intake when facing barriers to use the mobile phone dietary assessment app was an important facilitator.

SDT has been used in various areas of research [14]. For example, it has been used in the study of self-care in type 1 diabetes [15] and in relation to body image and unhealthy weight control behavior in adolescents [16]. However, to our knowledge, it has not been used in the study of dietary assessment methods. The results of the present study demonstrated that adolescents differed in their motivation; some participants appeared to be intrinsically motivated while others appeared to be controlled or autonomously extrinsically motivated. The most amotivated adolescents presumably chose not to participate in the study.

According to SDT, in order to enhance intrinsic motivation, the adolescents’ autonomy, competence, and relatedness in relation to the task of using the mobile phone dietary assessment app need to be supported. One way to achieve this goal is to plan studies that facilitate shared influence between participants and researchers (ie, with a participatory design). One study involving adolescents (before the current FR method was developed) found that participants preferred using mobile phones versus other methods of recording food intake [5]. Another study used participatory methods when developing text messages to improve nutrition and physical activity behaviors among teens [17]. However, the adolescents participating in these studies were not involved in formulating the research questions. It might not be feasible to involve adolescents in all steps of the research process. However, to feel that the research question is important to them, to be able to influence the way data are collected, and to be consulted in the interpretation process and the dissemination of results are all aspects that may increase the intrinsic motivation (ie, participate because the task is perceived as interesting or fun) [13]. Furthermore, in the present study, rewarding the controlled extrinsically motivated participants with a cinema ticket probably improved participation in this group, but the use of incentives likely reduced intrinsic motivation, since autonomy was thwarted [12].

One study that aimed to increase response rates in surveys showed that respondents were more satisfied with a questionnaire designed using SDT, and the response rates and data quality were higher compared with a standard questionnaire [18]. A limitation of the present study was that the mobile phone dietary assessment app was not designed using SDT. In future studies, by involving adolescents from the start, intrinsic motivation is likely to increase and there will be less need for rewards. Another advantage of involving adolescents in the design of the study and method development could be an increased potential to avoid two barriers that were identified in the present study (ie, uncertainty about what to record and what the study aim was). The number of practical barriers to use the method could also be reduced, further increasing the participants’ perceived competence. According to Ryan and Deci, consideration of the autonomy, competence, and relatedness of the participants may increase their motivation to complete an otherwise uninteresting task [12].

The identified barriers and facilitators for adolescents to record their diets with the mobile phone dietary assessment app need to be considered in future development of the method and research. Many of the categories point at practical barriers, and the method should be improved so that not only the most motivated adolescents manage to complete the FR. For example, the app should be developed for other operating systems than Android, and tested thoroughly to detect any technical problems before a study begins. A limitation of the present study was that the mobile phone dietary assessment app was only developed for Android. Some of the participants had problems using a type of mobile phone that they were not accustomed to, which likely distorted the perceived user-friendliness of the app. Very few studies have evaluated experiences using dietary assessment methods via qualitative methods. Vereecken et al used focus groups with children and parents to evaluate a web-based 24-hour recall method [19]. The children were enthusiastic about the method, but similar to the present study, some changes to improve the user-friendliness were requested (eg, regarding food items and reminders).

A study examining adult US women used focus groups to qualitatively investigate possible behavioral changes when keeping FRs [20]. The results showed that the participants altered their diets to include less snacks and more simple foods, because of the burden to complete the records. The women in the study also discussed the wish to report socially desirable foods, but claimed that they did not alter their recording. Conversely, children and adolescents said that they might change their dietary intake to make the recording process easier [6]. It is challenging to make adolescents want to keep an FR, and also to record data correctly, making it important to consider the effect that individual factors (such as social desirability) have on the results.

Qualitative methods are suitable when exploring experiences and views, and focus group interviews allow for self-disclosure among participants, and are suitable to shed light on quantitative data already collected [21]. When aiming to describe and interpret patterns in data, qualitative content analysis may be an appropriate technique [22]. Both focus group interviews and data analysis can be conducted by one researcher alone. However, focus groups often involve an observer, in addition to the researcher moderating the group [23]. Coding can be conducted by one researcher, although interrater subjectivity can be viewed as an approximation of objectivity [24]. Thus, we considered two research staff to be enough for both interviews and analysis in the present study.

Focus groups were chosen as the interview method because it was assumed that the method would provide rich data, as a result of interaction between the participants. Individual interviews have the advantage of decreasing the impact of peer pressure, allowing the participant to speak more freely. Even so, we assumed that the participants would be more comfortable being in a group interview, as opposed to talking to the interviewer and assistant alone. The interviews were held in separate rooms in the schools, aiming to make the participants feel comfortable. Each room was nearby in an environment well known to the students, and they were not disturbed by other students or teachers. The group interviews were held approximately two weeks after the participants had used the mobile phone dietary assessment app. We believe that this time was short enough for the participants to remember how they perceived the method, but long enough for them to have time to reflect.

The interviews did not last as long as anticipated, with an average of 19 minutes. However, there was not much small talk, and participants started to talk about the method almost immediately when entering the room. The participants might have found it difficult to focus on the interview since it was held in school and interrupted the usual schedule. During some interviews, the participants seemed restless and eager to get back to class. However, recruiting outside of school settings introduces other difficulties such as reaching adolescents from various backgrounds.

The present study included adolescents who had agreed to participate in an evaluation study of a mobile phone dietary assessment app. Although some participants did not finish the task, they were probably more motivated than those who choose not to participate in the study. Not all of the participants in the quantitative part of the evaluation study were interviewed. It was not possible for all teachers to assign class time on the second visit, and 17 of the 92 adolescents (who were in school-classes when initial group interviews were conducted) were not present at the time of the interview (eg, due to illness). This factor might imply that the adolescents who were interviewed were in better general health than those who were absent from school. This possible implication should be kept in mind when transferring results to other settings. It was the opinion of the researchers that data saturation was reached and that interviewing more participants would not change the results substantially.

The first and second author looked at the data separately, and after discussion agreed about the interpretation. The data were thoroughly discussed, from examples of meaning units and condensed meaning units, to labelling with codes and sorting responses into categories. The researchers further agreed about the theme, barriers and facilitators, and the application of SDT to the material. According to Graneheim and Lundman, these are important steps to achieve credibility of the research findings [11].

In conclusion, adolescents perceived several barriers, but also highlighted facilitators, when using a mobile phone dietary assessment app. Intersubject variations in the willingness to overcome the barriers could be understood by applying the different motivational types in SDT. Further research should aim at studying methods for (and effects of) increasing intrinsic motivation by supporting autonomy, competence, and relatedness among adolescents asked to participate in dietary studies. Hypothetically, such increased intrinsic motivation would decrease the negative impact of barriers, both on participation rate and quality of results. Participatory methods (ie, involving adolescents in decision making) may create potential for fewer barriers and more facilitators, further increasing the probability of obtaining valid FR.

## Appendix

Multimedia Appendix 1 Interview guide.

## Abbreviations

FR: food record
SDT: Self Determination Theory
