# Supplementary material for: Overcoming Barriers: Adolescents’ Experiences Using a Mobile Phone Dietary Assessment App
Source: JMIR Mhealth Uhealth. 2016 Jul 29;4(3):e92. doi: 10.2196/mhealth.5700 (PMC4982910; doi:10.2196/mhealth.5700)
Supplement: Multimedia Appendix 1 [file mhealth_v4i3e92_app1.pdf]

Interview guide

Introductory questions

Can you tell me what you were asked to do in the study?

What did you think it was like?

What was it like to use the app?

Key questions

Were you able to record everything you ate?

Were you able to answer the questions in the evening?

Did you look at the feedback?

Did you use it?

Did you change your eating because of the recording?

If so, why?

Was there anything you ate that was more difficult or burdensome to record?

Were there any situations when it was more difficult or inconvenient to record?

Is there anything in the application that can be improved?

What was it like to use the SenseWear Armband?

Did you complete everything (all three days)?

Why/why not?

If not, what could have made you complete everything?

What do you think is important when conducting a dietary study in the future?

Did participating in the study fulfil your expectations?

Concluding questions

Can you tell me what you think is most important of the things that we have been talking about?

Is there anything that you would like to add?
